# Butyric acid-producing bacterial spore levels in conventional raw milk vary by farm

**DOI:** 10.3168/jdsc.2022-0252

**Published:** 2022-11-18

**Authors:** X. Shi, C. Qian, S.I. Murphy, M. Wiedmann, N.H. Martin

**Affiliations:** Milk Quality Improvement Program, Department of Food Science, Cornell University, Ithaca, NY 14853

## Abstract

Anaerobic butyric acid-producing sporeforming bacteria (BAB) are important microbial contaminants in raw milk that may lead to premature spoilage of certain cheeses during aging. A study was conducted to determine the baseline levels of these spores in raw milk from 7 conventional Northeast United States dairy farms over a 1-yr period. The overall mean BAB concentration was 1.79 log_10_ most probable number per liter with spore levels differing significantly by farm. A post-hoc farm management practices survey was conducted to determine if there was an association between farm practices on BAB levels in raw milk from these farms. Survey questions included variables related to bedding, milking preparation procedures, teat and udder cleanliness scoring, holding area cleaning procedures, and udder clipping or flaming frequency. Each variable was fitted with a linear mixed-effects model, which revealed no significant association between farm-level factors and the initial BAB concentrations in raw milk; this finding was likely due to the small sample size in this study. To demonstrate the usefulness of our data beyond the initial baseline levels of BAB spores in raw milk, we used this data set to calculate minimum number of individual samples that would be needed to be collected in future studies, which was determined to be 96 to 126 samples, to evaluate the association between farm-level factors and BAB spore concentrations in raw milk. Overall, this study provides dairy industry stakeholders with baseline data on BAB spore levels in raw milk, along with a demonstration on how these data could be used in future studies to calculate sample sizes needed to assess the effect of farm management practices on BAB levels in raw milk.

## Highlights

• Baseline anaerobic butyric acid bacteria (BAB) spore levels in raw milk collected monthly for 1 year from 7 conventional dairy farms had a mean concentration of 1.79 log10 MPN/L.
• BAB levels varied significantly by farm.
• Analysis of a post-hoc farm management survey failed to identify any significant association between farm management practices and BAB spore levels in raw milk.

## Graphical Abstract

**Figure:**
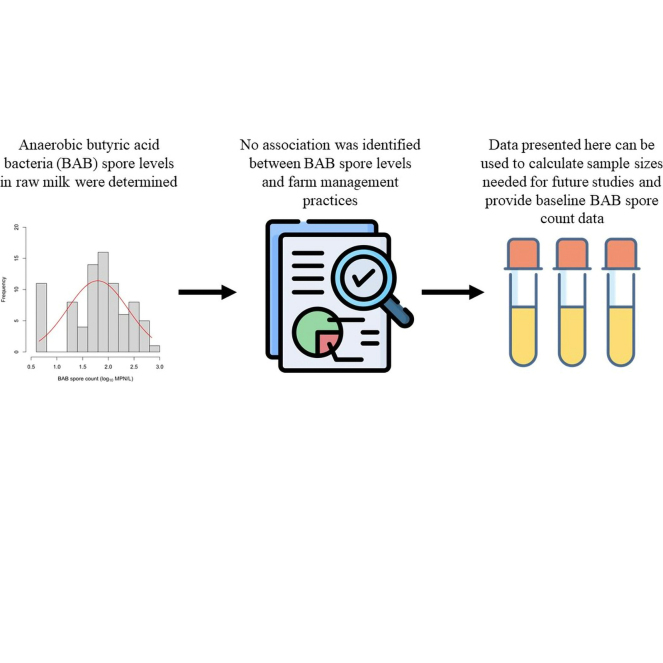
**Summary:** The levels of anaerobic butyric acid-producing sporeforming bacteria (BAB) in bulk tank raw milk from 7 similarly managed conventional dairy farms were studied. It is important to control and minimize BAB spores in raw milk to prevent late blowing defect in certain styles of cheese during aging. Different farm management practices such as bedding type used, cleaning agents, and udder clipping frequency may affect BAB spore concentrations in raw milk. We employed statistical models to test the relationship between farm-level factors and BAB spore concentrations. No significant association between management practices surveyed (i.e., bedding management, milking preparation procedures, teat and udder cleanliness scoring, holding area cleaning procedures, and udder clipping or flaming frequency) and BAB spore concentration was found, with one possible reason being the small sample size used. The minimum number of individual samples required was calculated using parameters from this study, demonstrating how this research can be used for future study design.

Anaerobic butyric acid-producing sporeforming bacteria (**BAB**) are naturally found in low levels in raw milk (e.g., 2.70 log_10_ spores/L reported by Vissers et al., 2007b) and are important to the dairy industry because some of these bacteria, namely *Clostridium tyrobutyricum* and related organisms, produce hydrogen gas and carbon dioxide during cheese ripening, resulting in cracks and splits in the cheese texture, a defect known as “late blowing” (Le Bourhis et al., 2007). In addition to cheese body defects resulting from gas production, organic acids such as butyric acid are produced during late blowing defect development in cheese, which is often accompanied by an unpleasant aroma and rancid flavor in the cheese product, thus causing consumer dissatisfaction (D'Incecco et al., 2018). This spoilage primarily affects semi-hard and hard cheese such as Gouda and Edam, resulting in major economic losses for cheese makers, and contributes to dairy food waste (Garde et al., 2013).

Previous studies have been conducted to assess methods for reducing late blowing defect in cheese at both the processing and farm levels (Waes and Heddeghem, 1990; Stadhouders, 1990; Velasco et al., 2011). Common measures to reduce BAB spore concentration in raw milk at the processing facility include mechanical removal of spores through bacterial centrifugation or filtration or preventing the outgrowth of BAB in cheese using additives such as lysozyme (Lodi and Stadhouders, 1990; Lamichhane et al., 2018). As spores of BAB in milk originate from farm sources (e.g., silage, bedding, manure, and so on), reducing or preventing contamination at the farm level is another viable method for reducing cheese spoilage due to BAB spores. Previous studies have examined the role that dairy cow feed plays in contamination levels of BAB spores in raw milk. For example, Vissers et al. (2007a) identified corn silage as the primary source of raw milk BAB spore contamination and indicated that preventing BAB spore levels from reaching high concentrations in the silage is critical to preventing subsequent BAB contamination of raw milk. In addition to feed sources, other studies have found that teat and udder hygiene are associated with BAB levels in bulk tank raw milk. In addition, Martin et al. (2019) identified udder hygiene as the most important farm management variable influencing BAB levels in bulk tank raw milk using a multimodel inference approach. However, most of these studies were performed in the Netherlands and production practices vary considerably between countries, limiting the application of these previous studies to US dairy farms. Therefore, our objective was to establish a baseline of BAB spore levels in raw milk from conventional dairy farms in the Northeast United States and, secondarily, to evaluate associations between these bulk tank raw milk BAB levels and farm management practices.

Raw milk was collected once per month from 7 conventional dairy farms (n = 12 samples from farms B, C, D, E, F, G and n = 11 samples from farm A) in the Northeast United States between July 2018 and June 2019 and tested for anaerobic BAB spores. Samples were collected from the bulk tank following the same procedures as outlined in Martin et al. (2019). Briefly, the bulk tank raw milk was agitated for 5 min before collecting a sample (~250 mL) using a dipper that had been sanitized in 0.2 mL/L chlorine. Samples were frozen at approximately −20°C before overnight shipment to the Milk Quality Improvement Program laboratory at Cornell University (Ithaca, NY). Upon receipt, frozen samples were stored at −20°C until microbiological testing occurred, typically within 1 wk.

Samples of frozen raw milk were thawed at 6°C for 24 h before testing for anaerobic BAB spores. Testing was performed using a most probable number (**MPN**) method as described in Martin et al. (2019) with modifications as follows. Briefly, raw milk samples were inoculated into a total of 20 sterile tubes of Bryant and Burkey (**BB**) media each, with 10 tubes receiving 5 mL of raw milk (inoculated into 5 mL of BB media) and 10 tubes receiving 500 µL of raw milk (inoculated into 9.5 mL of BB media). Tubes were capped with molten paraffin wax and heat treated at 75°C for 15 m to eliminate vegetative cells. All BB tubes were incubated at 35°C for 6 d and checked every 48 h for gas production, with tubes showing gas production (as indicated by movement of the wax plug) scored as positive and those with no gas production scored as negative.

A post-hoc farm management survey was conducted to identify potential factors associated with levels of BAB spores in bulk tank raw milk from these 7 farms (Table 1). The survey was modified from a previous survey used in Martin et al. (2019) and focused on (1) general farm-level information, including number of milking cows and how often each cow is milked per day; (2) bedding and housing area factors, including what type of bedding is used for lactating cows, how often is the bedding topped up, how often is the bedding dug out or changed, and how often alleyways are scraped per day; and (3) milking parlor factors, including whether or not gloves are worn during milking, whether or not cows are forestripped during the milking routine, whether or not laundered towels or paper towels are used during milking preparation, what cleaning methods are used for laundered towels, and whether or not the holding area is cleaned during milking, and if so how it is performed; and (4) cow level factors, including whether or not udder cleanliness is scored routinely, and if so how often, whether or not teat end cleanliness is scored routinely, and if so how often, and whether or not teat end condition is scored routinely, and if so how often. Variables with all the same responses, or with only one response dissimilar from the other responses, were not included in the analysis. These included alleyway scraping frequency and use of drying for laundered towels. To reduce collinearity in the data set and due to the relatively small sample size, the remaining 10 variables (after removal of redundant variables as described above) from the farm management survey (Table 1) were condensed into a total of 5 factors, representing (1) bedding (combination of bedding type and bedding topped up frequency); (2) holding area [combination of holding area cleaned (yes/no) and method (scraping or flushing) used to clean holding area]; (3) laundered towel cleaning protocol (combination of detergent use and bleach use); (4) udder clipping frequency; and (5) teat and udder scoring (combination of teat and udder cleanliness and condition scoring conducted and how often).

**Table 1:** Summary of farm management practice data collected from post-hoc surveys administered for 7 farms located throughout the Northeast United States

Farm	Approximate no. of milking cows	Bedding type^1^	Detergent used on towels^2^	Bleach used on towels^3^	Are towels dried?	Holding area scraped^4^	Holding area flushed^5^	Alleyways scraping frequency (daily)	Bedding topped up frequency (weekly)	Udders clipping frequency^6^ (yearly)	Udder cleanliness scoring frequency^7^	Teat end cleanliness scoring frequency^7^	Teat end condition scoring frequency^7^
A	1,820	Sand	No	Yes	No	No	Yes	3	2	4	Frequent	Not performed	Not performed
B	1,000	Sawdust	Yes	Yes	Yes	Yes	No	3	7	2	Not performed	Not performed	Not performed
C	1,700	Sand/sawdust^8^	Yes	No	Yes	Yes	No	1	2	2	Very frequent	Very frequent	Very frequent
D	1,250	Sand	Paper towel	Paper towel	Paper towel	Yes	Yes	3	1	4	Not performed	Not performed	Not performed
E	1,750	Sawdust	Yes	No	Yes	Yes	No	3	3	2	Infrequent	Infrequent	Infrequent
F	2,400	Manure solids	Yes	Yes	Yes	No	Yes	3	7	12	Frequent	Frequent	Not performed
G	1,650	Manure solids	Yes	Yes	Yes	Yes	Yes	3	6	4	Not performed	Frequent	Infrequent

1 Bedding type used for lactating cows.

2 Is detergent used on laundered towels used for milking preparation?

3 Is bleach used on towels used for milking preparation?

4 Is the holding area scraped during milking?

5 Is the holding area flushed with water during milking?

6 Are udders clipped or flamed?

7 Infrequent = scoring occurred 1 to 2 times per year; frequent = scoring occurred 3 to 12 times per year; very frequent = scoring occurred >12 times per year.

8 Approximately 50% of lactating cows were bedded on sand and approximately 50% on sawdust bedding.

All statistical analyses were performed in the R Statistical Programming Environment (R Core Team, 2020). Raw milk samples with BAB concentrations below the detection limit (18 MPN/L) were accounted for in the analyses by assigning a value of 25% of the detection limit (i.e., 4.5 MPN/L). Furthermore, one missing data point from farm A (October sample) was imputed by calculating the mean BAB count from the 11 BAB tests from that farm and assigning that value to the missing data point. Following data cleaning as described above, BAB spore concentrations were log_10_ transformed before further analysis. A total of 84 spore counts from 7 farms (A, B, C, D, E, F, G) were used in the statistical analysis. A frequency distribution of the BAB spore levels using all 84 data points was constructed by the histogram in R (R Core Team, 2020). Overall and pairwise differences between BAB spore levels by farm were evaluated using ANOVA using R built-in function (R 4.0.4) and Tukey's honestly significant difference using the emmeans package (version number 1.5.5–1).

To determine associations between the farm-level factors described above and BAB spore concentration in bulk tank raw milk the lme4 package in R was used (Bates et al., 2015) and individual linear mixed-effect models were fit for each of the 5 farm-level factors as a fixed effect, with log_10_ BAB spore count as the response. For each model, farm ID was included as a random effect to account for repeated measures from individual farms. Meteorological factors and season were not included in the analysis conducted here due to the limited sample size and as we had only one data point per farm/month combination, although no apparent trend was observed in the plotted data by farm over time. A *P*-value of 0.05 was used assess statistical significance for model outcomes.

Finally, to demonstrate the utility of the data presented here, the minimum number of individual samples needed to find an association between farm management practices and BAB spore levels in raw milk was calculated using the sample size techniques for descriptive studies outlined by Hulley et al. (2001). Briefly, the sample size calculation used 3 parameters: standard deviation of the variable of interest (**S**), the desired width of the interval (**W**) of the interval, and the confidence interval, where the value of W was determined based on individual study parameters. After standardizing the total width of the interval (dividing W by S), the sample size was determined according to the table presented in Hulley et al. (2001). In our study reported here, the confidence level was chosen to be 95% and the standardized total width of the interval (W/S) was between 0.35 and 0.40. Project data and code can be found at https://github.com/FSL-MQIP/BAB-Project.

Overall, BAB spore counts across the 7 farms approximately followed a normal distribution (Figure 1). Of the raw milk samples collected, 13.1% (11/84) had BAB spore levels below the limit of detection of 1.26 log_10_ MPN/L (18 MPN/L; Figure 1). Raw milk samples with detectable levels of BAB spores ranged from a low of 1.28 log_10_ MPN/L (19 MPN/L) to a high of 2.85 log_10_ MPN/L (700 MPN/L). The overall mean and standard error of the mean of BAB spore counts among the raw milk collected from the 7 farms was 1.79 ± 0.06 log_10_ MPN/L (62 ± 1 MPN/L). Mean spore levels varied widely and significantly (*P* < 0.0001) between individual farms. Based on arithmetic means, farm C had the lowest mean spore concentration of 1.40 log_10_ MPN/L (25.2 MPN/L), whereas farm E had the highest mean spore concentration across the 12 mo of 2.58 log_10_ MPN/L (378 MPN/L; Figure 2). Indeed, the mean spore level in raw milk from farm E was significantly higher (*P* < 0.0001) than the mean spore counts from each of the other farms (Figure 2). In addition to the higher mean spore levels, the range of spore counts for farm E [ranging from 2.18 to 2.85 log_10_ MPN/L (150 to 700 MPN/L)] was considerably larger than the range of spore counts in other farms (Figure 2). Limitations of our post-hoc survey (e.g., relying on producers to accurately recall changes in management practices over a year-long period) and lack of BAB data from potential farm sources (e.g., silage) limit our ability to determine the cause of the variability seen here; however, this variability may be attributed to changes in management practices such as milking preparation, due to level of BAB spores in feed or other farm-level factors. Previous studies have investigated the level of BAB spores in raw milk. For example, Vissers et al. (2007b) found the average BAB spore counts from 24 farms in the Netherlands to be 2.70 log_10_ spores/L, higher than the mean BAB spore count of 1.79 log_10_ MPN/L found in the current study. In addition to lower mean BAB spore concentrations in our study, none of the samples tested during this study exceeded the quality limit of 3.00 log_10_ BAB spores/L in raw milk that will be used for cheese making suggested by Vissers et al. (2007b). Further, while we observed significant differences between BAB spore concentrations in this study, other studies discussed here have not assessed if there are significant differences between BAB concentrations by farm or the level of variability within farm.

**Figure 1:**
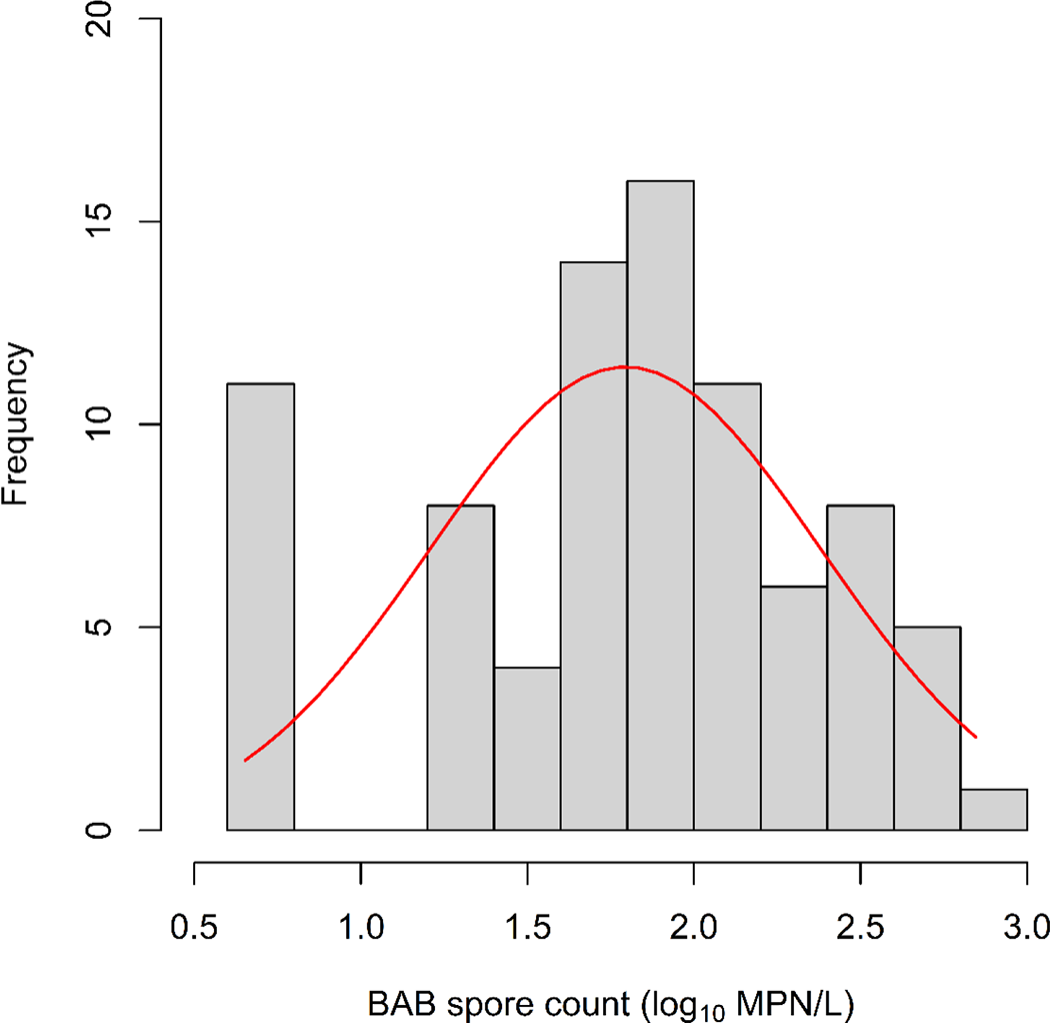
Overall anaerobic butyric acid-producing bacteria (BAB) spore count distribution from 84 raw milk samples collected from 7 farms in the Northeast United States over a 1-yr period. The red line represents the normal curve of total spore counts. MPN = most probable number.

**Figure 2:**
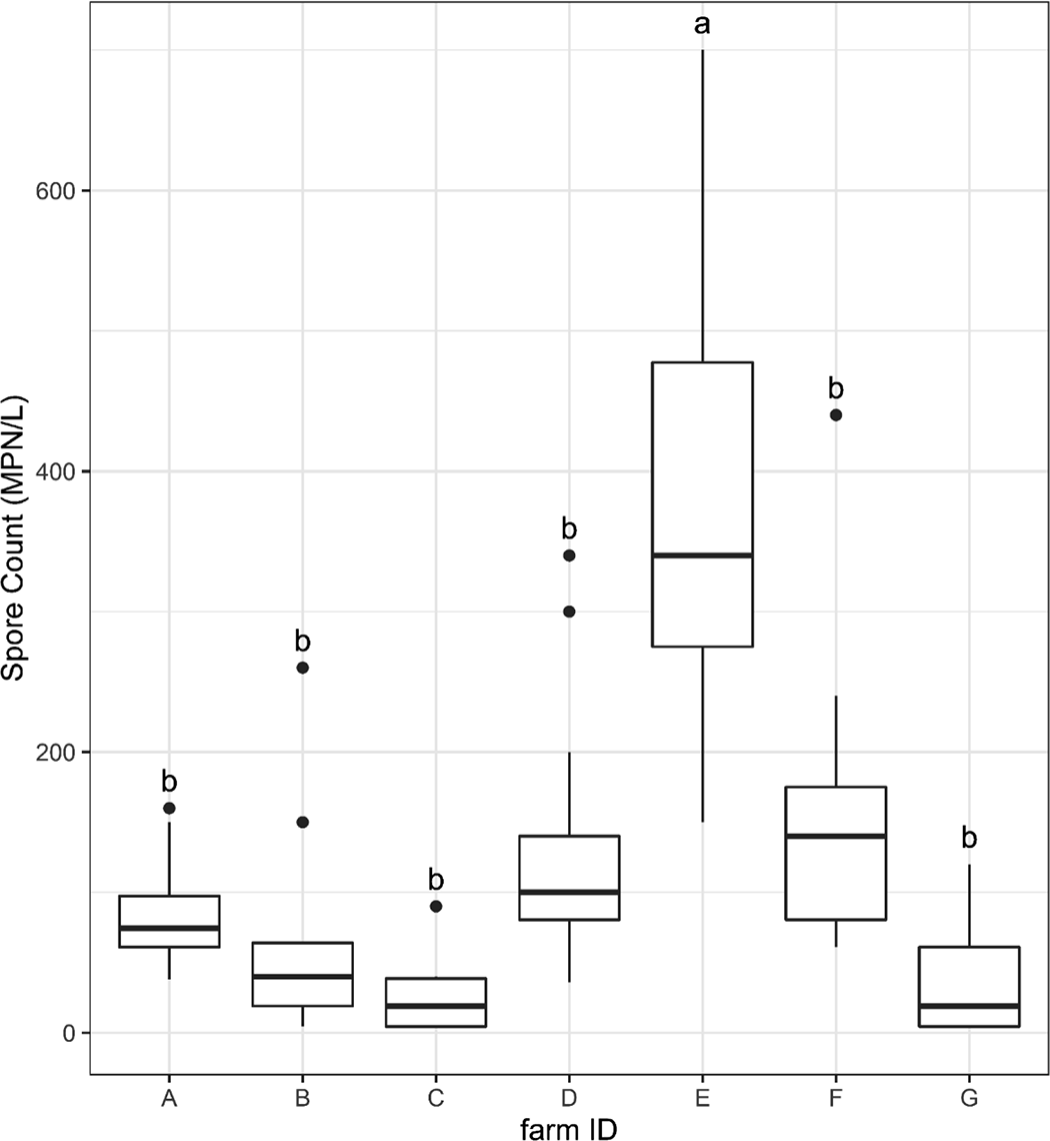
Anaerobic butyric acid-producing bacteria (BAB) spore count distribution by farm. The horizontal bars at the upper and lower whiskers of each box represent the maximum and minimum values, respectively. The horizontal bars in the middle of each box are the 50th percentile. The horizontal bars above and below the 50th percentile are the 75th and 25th percentiles, respectively. The overlaid dots represent outliers in each farm. Different letters (a, b) indicate significant differences (*P* < 0.0001; ANOVA) in BAB spore counts between individual farms. MPN = most probable number.

Overall, the approach used here showed that no farm-level factors assessed during this study were associated with BAB levels in raw milk, with *P*-values of holding area, cleaning agents, udder clipping frequency, and scoring models all greater than 0.8 and the *P*-value of the bedding model greater than 0.5 (data not shown). Previous studies have suggested that limiting the initial contamination of silage with BAB spores and preventing the growth of BAB during silage storage and feed-out is critical since the control of BAB spore levels in mixed silage was the most important strategy to control the spore concentration in raw milk (Vissers et al., 2007b). We attribute the nonsignificant results found in the current study in part to our limited sample size, consisting of 84 data points from 7 farms. To leverage the data collected from the current study for future research, we used the standard deviations calculated from this study to determine the minimum sample size needed to detect the association of farm-level factors and BAB spore concentrations. For example, using the 3 parameters outlined above and in Hulley et al. (2001), which includes (1) standard deviation (S), which was estimated here to be 0.59 log_10_ MPN/L (3.9 MPN/L); (2) standardized total width of the interval between 0.35 and 0.40 [calculated as the desired width of the interval (W) divided by S], and (3) the confidence interval of 95%, we estimate that a total of 96 to 126 samples would need to be tested to determine the impact of farm management practices on BAB spore levels in bulk tank raw milk. To calculate sample sizes needed to test individual farm management parameters (e.g., bedding type), future studies may employ standard deviations from this study, which can be found at https://github.com/FSL-MQIP/BAB-Project.

Our study provides a contemporary distribution of BAB spores in bulk tank raw milk in the Northeast United States, a valuable resource for the US dairy industry. While the number of samples here was a major limitation to our ability to determine associations between farm management practices and BAB spore levels in bulk tank raw milk, future studies should use the parameters calculated from our data set and the method demonstrated here to ensure a sufficient number of samples is collected. However, our data provide critical information on the significant variation of BAB spore counts between farms, which are valuable (and actionable) as they suggest that farm-level data on BAB concentrations can be used to identify farms that disproportionately contribute to BAB levels in comingled milk. Identifying these farms and reducing spore contamination represent an achievable strategy to reduce BAB spore concentrations in raw milk supplies used for cheesemaking, therefore reducing the likelihood of late blowing in cheese.
